# Complete Genome Sequence of the Cluster F1 Mycobacteriophage KingMidas

**DOI:** 10.1128/MRA.01557-19

**Published:** 2020-02-13

**Authors:** Christine A. Byrum, Christopher A. Korey, Zachary Jordan, Yang Zhou, Sarah Taylor, Joas Alfajardo, Veronique A. Delesalle

**Affiliations:** a Department of Biology, College of Charleston, Charleston, South Carolina, USA; b Department of Biology, Brown University, Providence, Rhode Island, USA; c Department of Biology, Gettysburg College, Gettysburg, Pennsylvania, USA; Queens College

## Abstract

Subcluster F1 bacteriophage KingMidas was isolated from soil collected in Providence, Rhode Island, using Mycobacterium smegmatis mc^2^155 as the host. The genome is 57,386 bp and contains 105 predicted protein-coding genes but no transfer-messenger RNAs or tRNAs. This siphovirus has an icosahedral head, with a genome 99.1% identical to that of F1 mycobacteriophage Scottish.

## ANNOUNCEMENT

**FIG 1:**
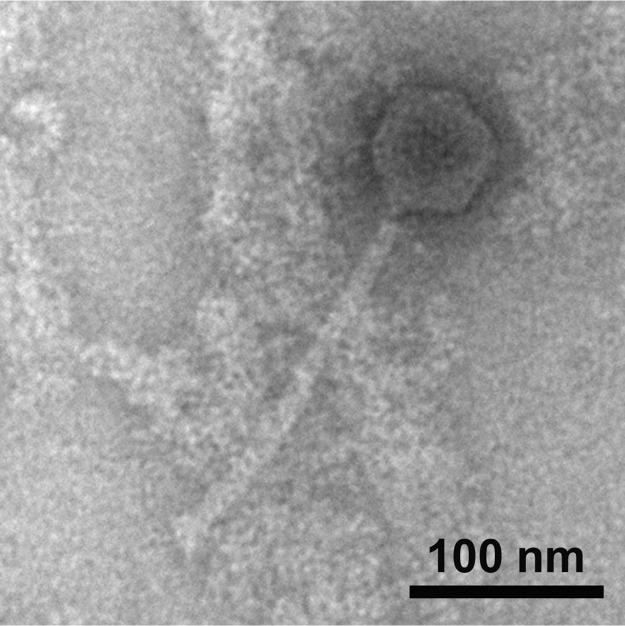
Morphology of the siphovirus KingMidas, examined using transmission electron microscopy. A high-titer lysate was deposited on Formvar-coated copper grids and negatively stained with 1% uranyl acetate.

KingMidas was obtained from soil under a sprinkler containing decomposing plants in Providence, Rhode Island (40.27018N, 97.12600W). In a Howard Hughes Medical Institute (HHMI) Science Education Alliance Phage Hunters Advancing Genomics and Evolutionary Science (SEA-PHAGES) (1) effort to characterize viral diversity and evolution, KingMidas was isolated (with enrichment at 23°C), purified, and amplified in Mycobacterium smegmatis mc^2^155, as described in the SEA-PHAGES Discovery Guide (https://seaphagesphagediscoveryguide.helpdocsonline.com/home). KingMidas forms clear plaques of 2.41 ± 0.10 mm (mean ± standard error) and possesses an icosahedral capsid (diameter, 61.12 ± 0.48 nm) with a flexible, noncontractile tail (length, 207.43 ± 2.08 nm; width, 12.11 ± 0.31 nm) (Fig. 1).

Following DNA extraction (Promega Wizard DNA clean-up system), sequencing libraries were produced (NEB Ultra II library kit). The Pittsburgh Bacteriophage Institute sequenced the DNA on an Illumina MiSeq system (v3 reagents) (2) to 1,672-fold coverage from 675,757 single-end reads (150 bp). Reads were quality trimmed and assembled *de novo* into a single contig using Newbler v2.9 (3), and the assembly was checked for completeness, accuracy, and genome termini using Consed v29.0 (2, 4). The 57,386-bp genome is linear, with cohesive 3′ overhangs (5′-CGGACGGCGC), a start site selected 108 bp upstream of the small terminase subunit, and a G+C content of 61.4%.

Annotation was performed using the PECAAN workflow tool (5), and completed files were transferred to DNA Master v5.22.23 (https://phagesdb.org/DNAMaster). PECAAN resources to identify putative genes included GLIMMER v3.0 (6), GeneMark v2.5 (7), Phamerator (8), Starterator v1.1 (https://seaphages.org/software), ARAGORN v1.2.38 (9), and tRNAscan-SE v3.0 (10). Functional assignments were made using BLASTp v2.9 (11), HHpred (12), TMHMM2 (http://www.cbs.dtu.dk/services/TMHMM), SOSUI v1.11 (13), and the NCBI Conserved Domain Database (14). Default settings were used for all programs.

KingMidas is a cluster F/subcluster F1 bacteriophage (15) with a genome containing 105 protein-coding genes (45 assigned putative functions) and no tRNAs or transfer-messenger RNAs. Like most mycobacteriophages (16), KingMidas has typical virion structural/assembly genes, including those encoding small and large terminase subunits, portal protein, capsid maturation protease, scaffolding protein, major capsid protein, head-to-tail adaptor, head-to-tail stopper, tail terminator, major tail protein, tail assembly chaperones, tape measure protein, and minor tail proteins. A lysis system is present (lysin A and lysin B), but the holin-like sequence (gp34) lacks transmembrane domains. KingMidas also includes genes for proteins that may regulate life cycle events (tyrosine integrase, excise protein, immunity repressor, Cro repressor, and antirepressor) and transcription (six helix-turn-helix DNA binding domain proteins and two WhiB-family transcription factors), as well as genes for two glycosyltransferases and proteins that may act in DNA synthesis, modification, or repair (DNAQ-like DNA polymerase III subunit, Ku-like double-stranded DNA break binding protein, three DNA methylases, an exonuclease, two HNH endonucleases, and RecA-like DNA recombinase). Rarely encountered F1 subcluster features (found in <10% of F1 genomes) include genes for a transposase (gp15) and an HNH endonuclease between lysin A and lysin B (gp32; truncated in KingMidas but longer in other F1 phages) and two genes with unknown function (gp102 and gp43).

Genome similarity was assessed using gene content similarity (GCS) scores (17) and BLASTn parameters. KingMidas is most similar to Scottish (GCS score, 93.2; identity, 99.1%; query coverage, 97%; GenBank accession number MN735433), an F1 bacteriophage found nearby (41.824167N, 71.403333W). KingMidas is also similar to Polka14 from Kansas (GCS score, 82.5; identity, 99.0%; query coverage, 93%; MK875792) and six bacteriophages from Pennsylvania, namely, Empress (GCS score, 88.9; identity, 97.6%; query coverage, 97%; KY012363), Nimbo (GCS score, 82.5; identity, 98.8%; query coverage, 92%; MH669009), Geralt (GCS score, 82.1; identity, 98.7%; query coverage, 92%; MF668271), PHappiness (GCS score, 81.2; identity, 99.3%; query coverage, 92%; MH669010), Daenerys (GCS score, 81.0; identity, 97.0%; query coverage, 92%; KF017005), and JoeyJr (GCS score, 77.2; identity, 97.1%; query coverage, 92%; MH669005), all of which are *Siphoviridae* F1 mycobacteriophages.

### Data availability.

The KingMidas genome is available in DDBJ/ENA/GenBank under accession number MN204502. The version described in this paper is the first version, MN204502.1. Raw reads appear in the SRA under accession number SRX6446236.
